# *CDKN2A/B* homozygous deletion is associated with early recurrence in meningiomas

**DOI:** 10.1007/s00401-020-02188-w

**Published:** 2020-07-08

**Authors:** Philipp Sievers, Thomas Hielscher, Daniel Schrimpf, Damian Stichel, David E. Reuss, Anna S. Berghoff, Marian C. Neidert, Hans-Georg Wirsching, Christian Mawrin, Ralf Ketter, Werner Paulus, Guido Reifenberger, Katrin Lamszus, Manfred Westphal, Nima Etminan, Miriam Ratliff, Christel Herold-Mende, Stefan M. Pfister, David T. W. Jones, Michael Weller, Patrick N. Harter, Wolfgang Wick, Matthias Preusser, Andreas von Deimling, Felix Sahm

**Affiliations:** 1 Department of Neuropathology, Institute of Pathology, University Hospital Heidelberg, Heidelberg, Germany; 2 Clinical Cooperation Unit Neuropathology (B300), German Consortium for Translational Cancer Research (DKTK), German Cancer Research Center (DKFZ), Heidelberg, Germany; 3 Division of Biostatistics, German Cancer Research Center (DKFZ), Heidelberg, Germany; 4 Institute of Neurology, Medical University of Vienna, Vienna, Austria; 5 Department of Neurosurgery, Clinical Neuroscience Center, University Hospital and University of Zurich, Zurich, Switzerland; 6 Department of Neurology, Clinical Neuroscience Center, University Hospital and University of Zurich, Zurich, Switzerland; 7 Department of Neuropathology, Otto-von-Guericke University, Magdeburg, Germany; 8 Department of Neurosurgery, University Hospital Homburg Saar, Homburg, Germany; 9 Institute of Neuropathology, University Hospital Münster, Münster, Germany; 10 Institute of Neuropathology, Heinrich Heine University, Düsseldorf, Germany; 11 German Cancer Consortium (DKTK), Partner Site Essen/Düsseldorf, Essen, Germany; 12 Department of Neurosurgery, University Medical Center Hamburg-Eppendorf, Hamburg, Germany; 13 Department of Neurosurgery, University Medical Centre Mannheim, University of Heidelberg, Mannheim, Germany; 14 Division of Experimental Neurosurgery, Department of Neurosurgery, University Hospital Heidelberg, Heidelberg, Germany; 15 Hopp Children’s Cancer Center Heidelberg (KiTZ), Heidelberg, Germany; 16 Division of Pediatric Neurooncology, German Cancer Consortium (DKTK), German Cancer Research Center (DKFZ), Heidelberg, Germany; 17 Department of Pediatric Oncology, Hematology, Immunology and Pulmonology, University Hospital Heidelberg, Heidelberg, Germany; 18 Pediatric Glioma Research Group, German Cancer Research Center (DKFZ), Heidelberg, Germany; 19 Institute of Neurology (Edinger Institute), Goethe University, Frankfurt, Germany; 20 German Cancer Consortium (DKTK), Partner Site Frankfurt/Mainz, Frankfurt am Main, Germany; 21 German Cancer Research Center (DKFZ), Heidelberg, Germany; 22 Frankfurt Cancer Institute (FCI), Frankfurt am Main, Germany; 23 Clinical Cooperation Unit Neurooncology, German Consortium for Translational Cancer Research (DKTK), German Cancer Research Center (DKFZ), Heidelberg, Germany; 24 Department of Neurology and Neurooncology Program, National Center for Tumor Diseases, Heidelberg University Hospital, Heidelberg, Germany; 25 Department of Medicine I, Clinical Division of Oncology and Comprehensive Cancer Center Vienna, Medical University of Vienna, Vienna, Austria

Most high-grade meningiomas show a highly perturbed copy number profile and they are enriched for *TERT* promoter mutations [6, 7, 11, 12]. In addition, homozygous focal deletions of the cyclin-dependent kinase inhibitor 2A (*CDKN2A*) gene, located at 9p21, have been observed at high frequency in anaplastic meningiomas [1, 3, 4, 9, 13]. An association of chromosome 9p21 deletion with malignant progression of meningiomas and poor prognosis, specifically in anaplastic meningiomas, has been demonstrated in 2002 by Perry et al. [9]. Here, we sought to determine the overall prognostic role of the *CDKN2A/B* status in a cohort of 528 meningioma patients with clinical follow-up data, covering all WHO grades and various subtypes. We thereby intended to assess the predictive power of the *CDKN2A/B* status, both independently and in the context of WHO grading, *TERT* promoter mutation status, and DNA methylation-based classification.

Tumor tissue and clinical follow-up data from 528 patients were obtained from the archives of multiple international collaborating centers and collected at the Department of Neuropathology, University Hospital Heidelberg (Heidelberg, Germany). Analysis of tissue and clinical data was performed in accordance with local ethical regulations. The clinical and pathologic characteristics of the study patients are summarized in Supplementary Table 1, online resource. DNA methylation profiling and copy number analysis of the tumors were performed using the Infinium MethylationEPIC (850k) BeadChip (Illumina, San Diego, CA, USA) or Infinium HumanMethylation450 (450k) BeadChip (Illumina) array as reported [2]. *TERT* promoter mutation status was assessed by Sanger sequencing or panel sequencing as previously described [5, 10]. Distribution of time to progression (as determined by imaging) or recurrence (TTP) after surgery was estimated by the Kaplan–Meier method and compared between groups with the log-rank test. Multivariable Cox proportional hazards regression was used to estimate the prognostic impact after adjusting for established prognostic factors. *p* values less than 0.05 were considered significant.

Among the tumors of 528 patients included in this study, 26 (4.9%) showed a homozygous deletion of *CDKN2A/B* as determined by DNA methylation array. Seven (27%) of these tumors were histologically graded as WHO grade II and 19 (73%) as WHO grade III. Notably, all tumors were either classified as atypical (constituting 4% of all atypical meningiomas) or anaplastic meningiomas (28% of all anaplastic meningiomas). Besides the most common grading criterion, proliferative activity, a set of morphological features also qualifies for WHO grade II or III according to the WHO classification, even in absence of high mitotic count. Interestingly, none of the WHO grade II or III meningioma variants diagnosed according to the proliferation-independent histological patterns, including chordoid, clear cell and rhabdoid meningiomas, showed a homozygous deletion of *CDKN2A/B* (Supplementary Table 1, online resource). In relation to the different methylation classes reported to independently stratify for risk of recurrence among meningioma [12], *CDKN2A/B* homozygous deletion was observed only in the methylation classes “intermediate” (*n* = 6; 23%) or “malignant” (*n* = 20; 77%). None of the tumors within the methylation class benign showed a homozygous deletion *of CDKN2A/B*. Of the 528 patients, 350 were female (66%) and the mean age at the time of surgery was 57 years (range 6–85 years). Median follow-up after surgery was 45 months (range 1–291 months), during which 175 patients had a progression or recurrence. Homozygous deletion of *CDKN2A/B* was neither significantly associated with patient age or sex nor with the tumor location. A detailed description of clinico-pathological characteristics is given in Supplementary Table 2, online resource.

Patients whose tumors carried *CDKN2A/B* homozygous deletions had a significantly worse outcome and more rapid progression from the time of surgery (*p* < 0.001; median TTP 8 vs. 101 months, Supplementary fig. 1, online resource). Importantly, this held true even within WHO grades (WHO grade II/III: *p* = 0.004/0.003; Fig. 1a, b). A significant difference was also observed within DNA methylation-based subtypes (intermediate/malignant: *p* = 0.03/0.02, correlation of these and other parameters depicted in Fig. 1c). *CDKN2A/B* status remained an independent prognostic factor in Cox regression when adjusting for WHO grade, DNA methylation-based classification, tumor location, age and sex (Supplementary Table 3, online resource).

**Fig. 1:**
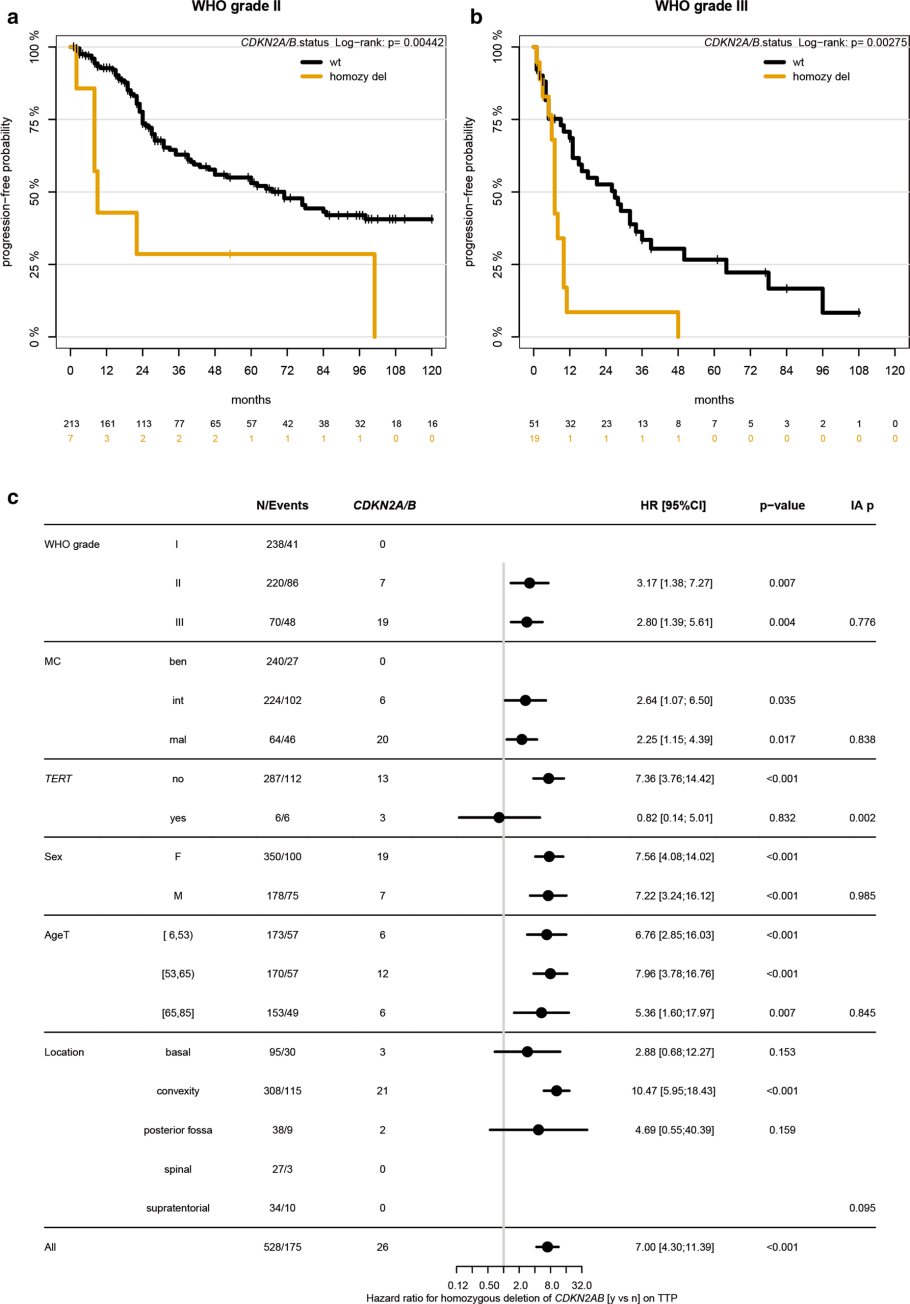
Time to progression or recurrence (TTP) of the 528 patients in the cohort stratified for *CDKN2A/B* homozygous deletion vs. WHO grade (**a**, **b**). Forest plot of univariable (unadjusted) hazard ratios for TTP for *CDKN2A/B* homozygous deletions stratified by WHO grade, methylation classes, *TERT* promoter mutation status, sex, age and location (**c**). AgeT gives age tertiles. IA *p* value gives *p* value for test on interaction, i.e., different prognostic effect of *CDKN2A/B* homozygous deletion in subgroups. *MC* methylation class, *ben* benign, *int* intermediate, *mal* malignant, *F* female, *M* male, *TTP* time to progression/recurrence

As *TERT* promoter mutations are associated with early recurrence [8, 11], we evaluated the effects of the *TERT* status on the TTP in a subset of patients (*n* = 293, 16 with *CDKN2A/B* homozygously deleted tumors) with available sequencing data, both individually and combined with the *CDKN2A/B* status. Tumors of 6/293 patients carried a *TERT* promoter mutation, three of them showed co-occurrence of a homozygous deletion of *CDKN2A/B* (*p* = 0.002). The outcome of patients with *TERT* promoter-mutant tumors was similarly unfavorable as that of patients with *CDKN2A/B* homozygously deleted tumors (median TTP for *TERT, CDKN2A/B* homozygous deletion, and other (i.e. none of both alterations): 11.5, 8, and 147 months, respectively; *TERT* status illustrated in Supplementary Fig. 2, online resource).

Our data demonstrates an independent adverse effect of *CDKN2A/B* homozygous deletion on the time to progression of patients with meningiomas. Thus, *CDKN2A/B* status can provide a useful biomarker for the identification of meningioma patients with a high risk of early recurrence. Consistent with previous studies, homozygous deletion of *CDKN2A/B* was found mainly in meningiomas graded as WHO grade II or III [1, 9, 13] which underlines a potential role in the malignant transformation of meningiomas. However, further studies will be needed for identification of the decisive steps in meningioma progression which will additionally assist in prioritizing the most relevant targets for novel therapy approaches. So far, *CDKN2A/B* homozygous deletion itself may be further clinically investigated as target for inhibitors of the CDK4/6 axis, e.g. ribociclib or palbociclib.

Notably, *CDKN2A/B* homozygous deletion allowed to further discriminate patients with unfavorable outcome within WHO grade II and III cases. This may suggest *CDKN2A/B* deletion as independent criterion for identification of highly aggressive (i.e. WHO grade III) meningiomas. However, a major limitation in the value of testing for *CDKN2A/B* homozygous deletion as well as *TERT* promoter mutation, both relevant to identify high-risk cases, is the low frequency of cases harboring such alterations. Furthermore, there seem to be a very small number of cases showing concordant alterations of *CDKN2A/B* and *TERT* which necessitates evaluation of both markers for a reliable risk prediction.

Interestingly, *CDKN2A/B* homozygous deletion further stratified for cases with highest risk of recurrence even within the methylation classes intermediate and malignant (Supplementary Fig. 3, online resource). This indicates that risk prediction based on methylation classes can be further refined when incorporating *CDKN2A/B* status, which will typically be available concurrently with generating methylation array data. Assessment of *CDKN2A/B* alone, however, cannot achieve the same prediction accuracy as methylation, since cases without homozygous deletion can still fall in any of the epigenetic classes.

In conclusion, our study demonstrates that homozygous deletion of *CDKN2A/B* is highly prognostic in meningiomas and may be a useful, independent molecular biomarker for grading of these tumors.

## Electronic supplementary material

Below is the link to the electronic supplementary material.

Supplementary material 1 (PDF 896 kb)
