# Supplementary material for: CDKN2A/B homozygous deletion is associated with early recurrence in meningiomas
Source: Acta Neuropathol. 2020 Jul 8;140(3):409–13. doi: 10.1007/s00401-020-02188-w (PMC7423850; doi:10.1007/s00401-020-02188-w)

Supplementary Methods

Sample collection. Tumor samples and clinical follow-up data from the 528 patients were obtained from the archives of multiple international collaborating centers and collected at the Department of Neuropathology, University Hospital Heidelberg (Heidelberg, Germany). Tissue sample collection and processing, data collection and use were performed in accordance with local ethics regulations and approvals.

DNA methylation array processing. Genomic DNA was extracted from fresh-frozen or formalin-fixed and paraffin-embedded (FFPE) tissue samples. The Illumina Infinium HumanMethylation450 (450k) array and Illumina Infinium MethylationEPIC (EPIC) array was used to obtain genome-wide DNA for tumor samples according to the manufacturer's instructions (Illumina, San Diego, CA, USA) and as previously described [1]. Data was generated at the Genomics and Proteomics Core Facility of the DKFZ (Heidelberg, Germany) or the Department of Neuropathology (Heidelberg, Germany). DNA methylation data were generated from both fresh-frozen and formalin-fixed paraffin-embedded (FFPE) tissue samples. For most fresh-frozen samples, >500ng of DNA was used as input material. 250ng of DNA was used for most FFPE tissues. All computational analyses were performed in R version 3.3.1 (R Development Core Team, 2016; https://www.R-project.org).

Determination of *TERT* promoter mutation status. *TERT* promoter mutation status was assessed by Sanger sequencing or next generation gene panel sequencing. Capture-based next-generation DNA sequencing was performed on a NextSeq 500 instrument (Illumina) as previously described [2] using a custom brain tumor panel covering the entire coding and selected intronic and promoter regions of 130 genes of particular relevance in central nervous system tumors. Reads were aligned against the reference genome (GRch37). Sanger sequencing was performed as described [3].

Statistical analysis. Fisher's exact test was used to compare categorical parameters. Wilcoxon Mann-Whitney test was used to compare the age distribution. Distribution of time to progression or recurrence (TTP) after surgery was estimated by the Kaplan-Meier method and compared between groups with the log-rank test. Multivariable Cox proportional hazards regression was used to estimate the prognostic impact after adjusting for established prognostic factors. For the multivariable Cox model missing value imputation was performed using the mice algorithm [4]. The likelihood-ratio test between the Cox model with and without interaction term of *CDKN2A/B* status and each clinico-pathological factor was used to determine potential subgroup effects of *CDKN2A/B* status (interaction p-value). P-values below 0.05 were considered significant.

References

1. Capper D, Jones DTW, Sill M, *et al.* DNA methylation-based classification of central nervous system tumours. Nature 2018;555(7697):469-474.
2. Sahm F, Schrimpf D, Jones DT, *et al.* Next-generation sequencing in routine brain tumor diagnostics enables an integrated diagnosis and identifies actionable targets. Acta Neuropathol 2016;131(6):903-10.
3. Koelsche C, Sahm F, Capper D, *et al.* Distribution of TERT promoter mutations in pediatric and adult tumors of the nervous system. Acta Neuropathol 2013;126(6):907-15.
4. Van Buuren S, Groothuis-Oudshoorn K. mice: Multivariate Imputation by Chained Equations in R. J Stat Softw 2011, (45):1–67.

Suppl. Table 1 – Association with clinico-pathological factors

	Level	wt	homozy del	p
n		502	26	
Age (median [IQR])		58.00 [48.00, 67.00]	59.00 [52.25, 65.00]	0.691
Sex (%)	F	331 (65.9)	19 (73.1)	0.528
	M	171 (34.1)	7 (26.9)	
Histology (%)	Meningothelial meningioma (WHO Grade I)	21 (4.2)	0 (0.0)	
	Fibroblastic meningioma (WHO Grade I)	28 (5.6)	0 (0.0)	
	Transitional meningioma (WHO Grade I)	65 (12.9)	0 (0.0)	
	Psammomatous meningioma (WHO grade I)	23 (4.6)	0 (0.0)	
	Angiomatous meningioma (WHO Grade I)	14 (2.8)	0 (0.0)	
	Microcystic meningioma (WHO grade I)	13 (2.6)	0 (0.0)	
	Secretory meningioma (WHO grade I)	24 (4.8)	0 (0.0)	
	Metaplastic meningioma (WHO Grade I)	8 (1.6)	0 (0.0)	
	Meningioma NOS	51 (10.2)	0 (0.0)	
	Chordoid meningioma (WHO grade II)	24 (4.8)	0 (0.0)	
	Clear cell meningioma (WHO grade II)	4 (0.8)	0 (0.0)	
	Atypical meningioma (WHO grade II)	176 (35.1)	7 (26.9)	
	Rhabdoid meningioma (WHO grade III)	1 (0.2)	0 (0.0)	
	Anaplastic meningioma (WHO grade III)	50 (10.0)	19 (73.1)	<0.001
Location (%)	basal	92 (19.3)	3 (11.5)	0.298
	convexity	287 (60.3)	21 (80.8)	
	posterior fossa	36 (7.6)	2 (7.7)	
	spinal	27 (5.7)	0 (0.0)	
	supratentorial	34 (7.1)	0 (0.0)	
WHO grade (%)	I	238 (47.4)	0 (0.0)	<0.001
	II	213 (42.4)	7 (26.9)	
	III	51 (10.2)	19 (73.1)	
MC (%)	ben	240 (47.8)	0 (0.0)	<0.001
	int	218 (43.4)	6 (23.1)	
	mal	44 (8.8)	20 (76.9)	
TERT (%)	no	274 (98.9)	13 (81.2)	0.002
	yes	3 (1.1)	3 (18.8)	
TERTvar (%)	C228T	1 (33.3)	2 (66.7)	1.000
	C250T	2 (66.7)	1 (33.3)	

Abbreviations: F – female, M – male, WHO - World Health Organization, MC – methylation class, ben – benign, int – intermediate, mal – malignant, wt – wildtype, homozy del - homozygous deletion, NOS - not otherwise specified. Meningioma NOS indicates that a specific subtyping of the meningioma was not available (inconclusive and/or material lacking for additional review).

Suppl. Table 2 – Clinico-pathological characteristics

Case #	Histology	WHO grade	Sex	Age (yrs)	PFS (m)	Progr./Rec.(1=yes;.0=no)	Location stand.	*CDKN2A/B* status	Methylation class	*TERT* promoter status
MNG_01	Anaplastic meningioma (WHO grade III)	3	M	49	3	1	convexity	homozy. del	mal	wt
MNG_02	Atypical meningioma (WHO grade II)	2	F	78	22	1	convexity	homozy. del	mal	wt
MNG_03	Atypical meningioma (WHO grade II)	2	F	61	9	1	convexity	homozy. del	mal	wt
MNG_04	Atypical meningioma (WHO grade II)	2	F	54	53	0	posterior fossa	homozy. del	int	wt
MNG_05	Anaplastic meningioma (WHO grade III)	3	F	22	7	1	posterior fossa	homozy. del	mal	wt
MNG_06	Anaplastic meningioma (WHO grade III)	3	M	58	48	1	convexity	homozy. del	mal	wt
MNG_07	Anaplastic meningioma (WHO grade III)	3	F	N/A	5	0	convexity	homozy. del	mal	wt
MNG_08	Anaplastic meningioma (WHO grade III)	3	M	48	5	1	convexity	homozy. del	mal	wt
MNG_09	Anaplastic meningioma (WHO grade III)	3	F	68	8	1	convexity	homozy. del	mal	wt
MNG_10	Anaplastic meningioma (WHO grade III)	3	M	53	6	1	convexity	homozy. del	mal	wt
MNG_11	Anaplastic meningioma (WHO grade III)	3	F	55	11	1	convexity	homozy. del	mal	N/A
MNG_12	Anaplastic meningioma (WHO grade III)	3	F	64	2	1	convexity	homozy. del	int	N/A
MNG_13	Anaplastic meningioma (WHO grade III)	3	F	61	1	0	convexity	homozy. del	mal	N/A
MNG_14	Anaplastic meningioma (WHO grade III)	3	F	74	5	0	basal	homozy. del	mal	N/A
MNG_15	Anaplastic meningioma (WHO grade III)	3	F	56	7	1	convexity	homozy. del	int	N/A
MNG_16	Anaplastic meningioma (WHO grade III)	3	F	56	1	1	convexity	homozy. del	int	N/A
MNG_17	Anaplastic meningioma (WHO grade III)	3	F	73	5	0	convexity	homozy. del	mal	N/A
MNG_18	Anaplastic meningioma (WHO grade III)	3	M	63	10	1	convexity	homozy. del	mal	N/A
MNG_19	Atypical meningioma (WHO grade II)	2	F	63	2	1	convexity	homozy. del	int	N/A
MNG_20	Anaplastic meningioma (WHO grade III)	3	F	60	3	0	convexity	homozy. del	mal	N/A
MNG_21	Atypical meningioma (WHO grade II)	2	F	50	101	1	basal	homozy. del	int	C250T
MNG_22	Atypical meningioma (WHO grade II)	2	F	36	8	1	basal	homozy. del	mal	wt
MNG_23	Atypical meningioma (WHO grade II)	2	M	N/A	8	1	convexity	homozy. del	mal	C228T
MNG_24	Anaplastic meningioma (WHO grade III)	3	F	70	10	1	convexity	homozy. del	mal	C228T
MNG_25	Anaplastic meningioma (WHO grade III)	3	F	83	1	0	convexity	homozy. del	mal	wt
MNG_26	Anaplastic meningioma (WHO grade III)	3	M	48	7	1	convexity	homozy. del	mal	wt
MNG_27	Fibroblastic meningioma (WHO Grade I)	1	F	63	12	0	supratentorial		ben	wt
MNG_28	Atypical meningioma (WHO grade II)	2	F	59	106	0	convexity		int	wt
MNG_29	Chordoid meningioma (WHO grade II)	2	F	41	20	1	convexity		int	wt
MNG_30	Chordoid meningioma (WHO grade II)	2	M	63	29	0	basal		ben	wt
MNG_31	Chordoid meningioma (WHO grade II)	2	F	51	3	0	basal		ben	N/A
MNG_32	Chordoid meningioma (WHO grade II)	2	F	51	19	1	convexity		int	wt
MNG_33	Chordoid meningioma (WHO grade II)	2	F	58	77	1	supratentorial		int	wt
MNG_34	Chordoid meningioma (WHO grade II)	2	F	47	96	0	convexity		ben	wt
MNG_35	Transitional meningioma (WHO Grade I)	1	M	38	59	0	convexity		int	wt

MNG_36	Metaplastic meningioma (WHO Grade I)	1	M	79	34	0	convexity		ben	wt
MNG_37	Transitional meningioma (WHO Grade I)	1	F	59	36	0	basal		ben	wt
MNG_38	Transitional meningioma (WHO Grade I)	1	F	47	1	0	spinal		ben	wt
MNG_39	Transitional meningioma (WHO Grade I)	1	F	75	3	0	spinal		int	wt
MNG_40	Anaplastic meningioma (WHO grade III)	3	F	79	34	0	convexity		int	wt
MNG_41	Transitional meningioma (WHO Grade I)	1	F	15	46	0	basal		ben	N/A
MNG_42	Secretory meningioma (WHO grade I)	1	F	55	17	0	supratentorial		ben	wt
MNG_43	Atypical meningioma (WHO grade II)	2	F	33	15	1	posterior fossa		mal	wt
MNG_44	Angiomatous meningioma (WHO Grade I)	1	F	67	39	0	supratentorial		ben	wt
MNG_45	Atypical meningioma (WHO grade II)	2	F	44	92	0	convexity		int	N/A
MNG_46	Atypical meningioma (WHO grade II)	2	F	64	102	0	supratentorial		int	wt
MNG_47	Transitional meningioma (WHO Grade I)	1	M	16	68	0	spinal		ben	N/A
MNG_48	Atypical meningioma (WHO grade II)	2	M	65	9	1	convexity		int	wt
MNG_49	Atypical meningioma (WHO grade II)	2	F	53	43	1	convexity		ben	wt
MNG_50	Atypical meningioma (WHO grade II)	2	F	35	71	1	basal		int	wt
MNG_51	Atypical meningioma (WHO grade II)	2	M	56	60	1	convexity		int	wt
MNG_52	Atypical meningioma (WHO grade II)	2	F	58	26	1	convexity		mal	wt
MNG_53	Atypical meningioma (WHO grade II)	2	M	56	3	1	convexity		int	wt
MNG_54	Atypical meningioma (WHO grade II)	2	M	74	35	1	convexity		int	wt
MNG_55	Atypical meningioma (WHO grade II)	2	F	68	31	1	convexity		int	wt
MNG_56	Atypical meningioma (WHO grade II)	2	M	36	31	1	convexity		int	wt
MNG_57	Fibroblastic meningioma (WHO Grade I)	1	F	53	100	0	N/A		int	wt
MNG_58	Atypical meningioma (WHO grade II)	2	F	54	100	0	N/A		int	wt
MNG_59	Atypical meningioma (WHO grade II)	2	F	62	18	1	convexity		int	wt
MNG_60	Atypical meningioma (WHO grade II)	2	F	48	100	0	convexity		ben	wt
MNG_61	Atypical meningioma (WHO grade II)	2	M	48	100	0	N/A		int	wt
MNG_62	Atypical meningioma (WHO grade II)	2	M	36	65	1	convexity		int	wt
MNG_63	Atypical meningioma (WHO grade II)	2	M	63	33	1	convexity		int	wt
MNG_64	Atypical meningioma (WHO grade II)	2	M	64	3	1	convexity		int	wt
MNG_65	Atypical meningioma (WHO grade II)	2	M	64	67	1	convexity		int	wt
MNG_66	Chordoid meningioma (WHO grade II)	2	F	63	35	1	convexity		int	wt
MNG_67	Transitional meningioma (WHO Grade I)	1	F	69	18	1	convexity		int	wt
MNG_68	Atypical meningioma (WHO grade II)	2	F	58	62	1	convexity		int	wt
MNG_69	Atypical meningioma (WHO grade II)	2	F	45	25	1	convexity		int	wt
MNG_70	Atypical meningioma (WHO grade II)	2	M	65	9	1	convexity		int	wt
MNG_71	Anaplastic meningioma (WHO grade III)	3	F	66	4	1	convexity		mal	wt
MNG_72	Transitional meningioma (WHO Grade I)	1	F	55	36	1	basal		ben	wt
MNG_73	Anaplastic meningioma (WHO grade III)	3	F	33	96	1	convexity		int	wt

MNG_74	Metaplastic meningioma (WHO Grade I)	1	M	53	120	0	convexity		ben	wt
MNG_75	Atypical meningioma (WHO grade II)	2	F	37	170	0	convexity		ben	wt
MNG_76	Fibroblastic meningioma (WHO Grade I)	1	F	45	137	0	convexity		ben	wt
MNG_77	Secretory meningioma (WHO grade I)	1	M	51	145	0	basal		ben	wt
MNG_78	Transitional meningioma (WHO Grade I)	1	F	62	85	0	posterior fossa		ben	wt
MNG_79	Angiomatous meningioma (WHO Grade I)	1	M	54	126	0	basal		ben	wt
MNG_80	Secretory meningioma (WHO grade I)	1	F	57	130	0	basal		ben	wt
MNG_81	Atypical meningioma (WHO grade II)	2	F	58	147	1	convexity		ben	wt
MNG_82	Fibroblastic meningioma (WHO Grade I)	1	F	44	3	1	convexity		int	wt
MNG_83	Atypical meningioma (WHO grade II)	2	F	16	22	1	basal		int	wt
MNG_84	Atypical meningioma (WHO grade II)	2	F	41	108	0	convexity		int	wt
MNG_85	Fibroblastic meningioma (WHO Grade I)	1	F	63	73	0	basal		ben	wt
MNG_86	Chordoid meningioma (WHO grade II)	2	F	68	17	1	convexity		ben	wt
MNG_87	Transitional meningioma (WHO Grade I)	1	F	N/A	96	0	basal		ben	wt
MNG_88	Transitional meningioma (WHO Grade I)	1	M	47	39	1	basal		int	N/A
MNG_89	Transitional meningioma (WHO Grade I)	1	F	51	103	0	supratentorial		ben	wt
MNG_90	Fibroblastic meningioma (WHO Grade I)	1	F	59	115	0	convexity		ben	wt
MNG_91	Atypical meningioma (WHO grade II)	2	F	49	12	0	convexity		int	wt
MNG_92	Transitional meningioma (WHO Grade I)	1	F	64	110	0	convexity		int	wt
MNG_93	Secretory meningioma (WHO grade I)	1	M	49	78	0	convexity		ben	wt
MNG_94	Transitional meningioma (WHO Grade I)	1	F	N/A	33	1	convexity		int	wt
MNG_95	Transitional meningioma (WHO Grade I)	1	F	N/A	20	1	basal		ben	wt
MNG_96	Fibroblastic meningioma (WHO Grade I)	1	F	44	50	0	convexity		ben	wt
MNG_97	Transitional meningioma (WHO Grade I)	1	F	59	85	1	posterior fossa		ben	wt
MNG_98	Meningothelial meningioma (WHO Grade I)	1	F	60	49	0	basal		ben	wt
MNG_99	Transitional meningioma (WHO Grade I)	1	M	52	54	1	convexity		int	wt
MNG_100	Meningothelial meningioma (WHO Grade I)	1	M	54	101	1	convexity		int	wt
MNG_101	Transitional meningioma (WHO Grade I)	1	F	N/A	108	1	convexity		int	wt
MNG_102	Fibroblastic meningioma (WHO Grade I)	1	M	52	85	1	posterior fossa		int	wt
MNG_103	Transitional meningioma (WHO Grade I)	1	M	71	111	0	convexity		int	wt
MNG_104	Atypical meningioma (WHO grade II)	2	M	N/A	132	0	convexity		int	wt
MNG_105	Atypical meningioma (WHO grade II)	2	F	83	107	0	convexity		ben	wt
MNG_106	Atypical meningioma (WHO grade II)	2	F	N/A	125	0	convexity		ben	wt
MNG_107	Atypical meningioma (WHO grade II)	2	F	49	98	0	convexity		ben	wt
MNG_108	Atypical meningioma (WHO grade II)	2	F	N/A	156	0	convexity		ben	wt
MNG_109	Anaplastic meningioma (WHO grade III)	3	F	63	77	0	convexity		ben	wt
MNG_110	Atypical meningioma (WHO grade II)	2	F	N/A	141	0	convexity		ben	wt
MNG_111	Atypical meningioma (WHO grade II)	2	F	N/A	120	0	convexity		ben	wt

MNG_112	Atypical meningioma (WHO grade II)	2	F	N/A	24	1	convexity		int	wt
MNG_113	Atypical meningioma (WHO grade II)	2	M	N/A	19	1	convexity		int	wt
MNG_114	Atypical meningioma (WHO grade II)	2	F	26	100	0	convexity		ben	wt
MNG_115	Atypical meningioma (WHO grade II)	2	F	54	14	1	convexity		int	wt
MNG_116	Atypical meningioma (WHO grade II)	2	M	64	19	1	posterior fossa		int	wt
MNG_117	Atypical meningioma (WHO grade II)	2	M	71	23	1	convexity		int	wt
MNG_118	Atypical meningioma (WHO grade II)	2	M	63	22	1	convexity		int	wt
MNG_119	Fibroblastic meningioma (WHO Grade I)	1	F	56	133	0	convexity		ben	wt
MNG_120	Anaplastic meningioma (WHO grade III)	3	M	39	39	1	basal		int	wt
MNG_121	Anaplastic meningioma (WHO grade III)	3	F	58	50	1	convexity		ben	wt
MNG_122	Transitional meningioma (WHO Grade I)	1	M	47	72	0	convexity		int	wt
MNG_123	Transitional meningioma (WHO Grade I)	1	F	68	128	0	basal		int	wt
MNG_124	Atypical meningioma (WHO grade II)	2	F	65	27	1	basal		int	wt
MNG_125	Meningioma NOS	1	F	48	180	1	N/A		ben	wt
MNG_126	Meningothelial meningioma (WHO Grade I)	1	F	35	192	1	convexity		ben	wt
MNG_127	Atypical meningioma (WHO grade II)	2	F	39	24	1	convexity		int	wt
MNG_128	Atypical meningioma (WHO grade II)	2	F	68	40	1	convexity		int	wt
MNG_129	Meningothelial meningioma (WHO Grade I)	1	M	36	19	0	convexity		int	wt
MNG_130	Meningothelial meningioma (WHO Grade I)	1	F	39	156	1	basal		ben	wt
MNG_131	Atypical meningioma (WHO grade II)	2	F	71	39	1	supratentorial		int	wt
MNG_132	Secretory meningioma (WHO grade I)	1	F	44	92	0	convexity		ben	wt
MNG_133	Atypical meningioma (WHO grade II)	2	M	41	19	1	convexity		int	wt
MNG_134	Microcystic meningioma (WHO grade I)	1	F	53	91	0	basal		ben	wt
MNG_135	Microcystic meningioma (WHO grade I)	1	F	64	78	0	convexity		int	wt
MNG_136	Meningioma NOS	1	F	47	102	1	basal		ben	wt
MNG_137	Anaplastic meningioma (WHO grade III)	3	M	52	21	1	convexity		int	wt
MNG_138	Psammomatous meningioma (WHO grade I)	1	F	66	123	0	posterior fossa		int	wt
MNG_139	Atypical meningioma (WHO grade II)	2	M	68	68	0	convexity		int	wt
MNG_140	Psammomatous meningioma (WHO grade I)	1	F	70	131	0	basal		ben	wt
MNG_141	Fibroblastic meningioma (WHO Grade I)	1	F	56	129	0	posterior fossa		ben	wt
MNG_142	Anaplastic meningioma (WHO grade III)	3	M	N/A	7	0	basal		int	wt
MNG_143	Anaplastic meningioma (WHO grade III)	3	M	N/A	29	1	convexity		int	wt
MNG_144	Atypical meningioma (WHO grade II)	2	M	79	89	0	convexity		int	wt
MNG_145	Meningothelial meningioma (WHO Grade I)	1	M	52	60	1	convexity		int	wt
MNG_146	Atypical meningioma (WHO grade II)	2	F	35	8	1	convexity		mal	wt
MNG_147	Angiomatous meningioma (WHO Grade I)	1	M	43	138	0	convexity		ben	wt
MNG_148	Atypical meningioma (WHO grade II)	2	M	60	84	1	basal		int	wt
MNG_149	Atypical meningioma (WHO grade II)	2	M	13	3	1	convexity		int	wt

MNG_150	Meningioma NOS	2	F	57	106	0	posterior fossa		ben	wt
MNG_151	Microcystic meningioma (WHO grade I)	1	M	63	108	0	basal		ben	wt
MNG_152	Meningioma NOS	2	M	44	52	1	N/A		mal	wt
MNG_153	Anaplastic meningioma (WHO grade III)	3	F	62	18	1	convexity		mal	wt
MNG_154	Meningioma NOS	2	F	60	27	0	posterior fossa		ben	wt
MNG_155	Psammomatous meningioma (WHO grade I)	1	M	61	111	0	convexity		ben	wt
MNG_156	Secretory meningioma (WHO grade I)	1	F	75	123	0	basal		ben	wt
MNG_157	Meningioma NOS	2	M	72	99	0	convexity		int	wt
MNG_158	Meningioma NOS	2	F	64	126	0	convexity		int	wt
MNG_159	Meningioma NOS	2	F	50	95	0	basal		ben	wt
MNG_160	Meningioma NOS	2	M	77	111	0	posterior fossa		int	wt
MNG_161	Psammomatous meningioma (WHO grade I)	1	F	70	143	0	posterior fossa		ben	wt
MNG_162	Psammomatous meningioma (WHO grade I)	1	F	61	125	0	basal		int	wt
MNG_163	Atypical meningioma (WHO grade II)	2	M	66	24	1	convexity		int	wt
MNG_164	Angiomatous meningioma (WHO Grade I)	1	F	42	146	0	convexity		ben	wt
MNG_165	Chordoid meningioma (WHO grade II)	2	F	76	39	0	convexity		int	wt
MNG_166	Fibroblastic meningioma (WHO Grade I)	1	F	42	132	0	convexity		ben	wt
MNG_167	Angiomatous meningioma (WHO Grade I)	1	M	38	166	0	convexity		ben	wt
MNG_168	Fibroblastic meningioma (WHO Grade I)	1	F	72	39	0	convexity		ben	wt
MNG_169	Anaplastic meningioma (WHO grade III)	3	M	38	27	1	basal		int	wt
MNG_170	Atypical meningioma (WHO grade II)	2	F	66	39	0	posterior fossa		int	wt
MNG_171	Meningioma NOS	2	M	39	48	1	N/A		int	wt
MNG_172	Anaplastic meningioma (WHO grade III)	3	F	79	2	1	N/A		mal	wt
MNG_173	Meningothelial meningioma (WHO Grade I)	1	F	77	17	0	supratentorial		int	wt
MNG_174	Anaplastic meningioma (WHO grade III)	3	F	57	3	1	convexity		mal	wt
MNG_175	Angiomatous meningioma (WHO Grade I)	1	F	53	89	0	convexity		ben	wt
MNG_176	Atypical meningioma (WHO grade II)	2	F	33	28	1	convexity		ben	wt
MNG_177	Anaplastic meningioma (WHO grade III)	3	M	78	64	1	convexity		int	wt
MNG_178	Atypical meningioma (WHO grade II)	2	M	67	66	0	convexity		int	wt
MNG_179	Chordoid meningioma (WHO grade II)	2	F	52	100	0	convexity		ben	wt
MNG_180	Meningioma NOS	1	M	54	35	0	basal		ben	N/A
MNG_181	Atypical meningioma (WHO grade II)	2	F	30	2	1	convexity		int	wt
MNG_182	Meningioma NOS	2	M	61	98	1	convexity		int	wt
MNG_183	Microcystic meningioma (WHO grade I)	1	F	36	40	0	convexity		ben	wt
MNG_184	Transitional meningioma (WHO Grade I)	1	M	46	35	0	basal		int	N/A
MNG_185	Secretory meningioma (WHO grade I)	1	F	58	1	0	basal		ben	N/A
MNG_186	Secretory meningioma (WHO grade I)	1	F	39	48	0	convexity		ben	N/A
MNG_187	Psammomatous meningioma (WHO grade I)	1	F	52	29	1	spinal		int	N/A

MNG_188	Anaplastic meningioma (WHO grade III)	3	M	83	13	1	convexity		int	N/A
MNG_189	Microcystic meningioma (WHO grade I)	1	M	42	104	0	N/A		ben	N/A
MNG_190	Atypical meningioma (WHO grade II)	2	M	59	38	0	convexity		int	N/A
MNG_191	Chordoid meningioma (WHO grade II)	2	F	50	28	1	convexity		mal	N/A
MNG_192	Psammomatous meningioma (WHO grade I)	1	F	67	28	0	convexity		ben	N/A
MNG_193	Psammomatous meningioma (WHO grade I)	1	M	64	13	0	spinal		ben	N/A
MNG_194	Secretory meningioma (WHO grade I)	1	F	51	32	0	posterior fossa		ben	N/A
MNG_195	Secretory meningioma (WHO grade I)	1	F	57	109	0	basal		ben	N/A
MNG_196	Psammomatous meningioma (WHO grade I)	1	F	43	45	0	basal		ben	N/A
MNG_197	Psammomatous meningioma (WHO grade I)	1	F	80	2	0	spinal		ben	N/A
MNG_198	Psammomatous meningioma (WHO grade I)	1	F	67	50	0	basal		int	N/A
MNG_199	Anaplastic meningioma (WHO grade III)	3	M	64	3	0	basal		ben	N/A
MNG_200	Atypical meningioma (WHO grade II)	2	F	64	85	1	basal		int	N/A
MNG_201	Atypical meningioma (WHO grade II)	2	M	56	16	1	convexity		int	N/A
MNG_202	Secretory meningioma (WHO grade I)	1	F	66	40	0	convexity		ben	N/A
MNG_203	Secretory meningioma (WHO grade I)	1	F	51	15	0	basal		ben	N/A
MNG_204	Secretory meningioma (WHO grade I)	1	F	57	41	0	convexity		ben	N/A
MNG_205	Psammomatous meningioma (WHO grade I)	1	F	55	2	0	basal		ben	N/A
MNG_206	Psammomatous meningioma (WHO grade I)	1	F	73	2	0	spinal		int	N/A
MNG_207	Anaplastic meningioma (WHO grade III)	3	F	64	36	0	posterior fossa		ben	N/A
MNG_208	Atypical meningioma (WHO grade II)	2	M	50	26	0	convexity		int	N/A
MNG_209	Atypical meningioma (WHO grade II)	2	F	76	3	0	convexity		ben	N/A
MNG_210	Atypical meningioma (WHO grade II)	2	F	45	7	0	convexity		int	N/A
MNG_211	Atypical meningioma (WHO grade II)	2	F	69	8	0	convexity		int	N/A
MNG_212	Psammomatous meningioma (WHO grade I)	1	M	20	19	1	spinal		int	N/A
MNG_213	Anaplastic meningioma (WHO grade III)	3	F	64	61	0	convexity		int	N/A
MNG_214	Atypical meningioma (WHO grade II)	2	M	52	9	0	posterior fossa		ben	N/A
MNG_215	Chordoid meningioma (WHO grade II)	2	F	55	26	0	N/A		ben	N/A
MNG_216	Anaplastic meningioma (WHO grade III)	3	M	68	5	1	N/A		mal	N/A
MNG_217	Atypical meningioma (WHO grade II)	2	M	61	73	0	convexity		int	N/A
MNG_218	Secretory meningioma (WHO grade I)	1	F	38	29	0	basal		ben	N/A
MNG_219	Meningioma NOS	1	M	62	6	0	convexity		ben	N/A
MNG_220	Psammomatous meningioma (WHO grade I)	1	F	48	22	1	spinal		int	N/A
MNG_221	Psammomatous meningioma (WHO grade I)	1	F	63	17	0	spinal		ben	N/A
MNG_222	Anaplastic meningioma (WHO grade III)	3	M	61	32	0	convexity		int	N/A
MNG_223	Anaplastic meningioma (WHO grade III)	3	M	66	15	1	convexity		mal	N/A
MNG_224	Chordoid meningioma (WHO grade II)	2	F	47	61	0	convexity		ben	N/A
MNG_225	Chordoid meningioma (WHO grade II)	2	F	51	16	0	convexity		ben	N/A

MNG_226	Chordoid meningioma (WHO grade II)	2	F	52	69	0	basal		ben	N/A
MNG_227	Meningioma NOS	1	F	48	1	0	convexity		ben	N/A
MNG_228	Anaplastic meningioma (WHO grade III)	3	F	68	1	1	basal		mal	N/A
MNG_229	Secretory meningioma (WHO grade I)	1	F	67	42	0	basal		ben	N/A
MNG_230	Atypical meningioma (WHO grade II)	2	M	6	71	0	N/A		ben	N/A
MNG_231	Atypical meningioma (WHO grade II)	2	F	69	41	1	convexity		int	N/A
MNG_232	Atypical meningioma (WHO grade II)	2	M	43	4	0	basal		ben	N/A
MNG_233	Microcystic meningioma (WHO grade I)	1	F	47	23	1	basal		ben	N/A
MNG_234	Microcystic meningioma (WHO grade I)	1	F	49	44	1	basal		ben	N/A
MNG_235	Chordoid meningioma (WHO grade II)	2	F	51	30	0	basal		ben	N/A
MNG_236	Microcystic meningioma (WHO grade I)	1	F	39	5	1	N/A		ben	N/A
MNG_237	Anaplastic meningioma (WHO grade III)	3	F	63	32	1	convexity		int	N/A
MNG_238	Atypical meningioma (WHO grade II)	2	M	72	62	0	convexity		int	N/A
MNG_239	Atypical meningioma (WHO grade II)	2	F	59	24	1	convexity		int	N/A
MNG_240	Chordoid meningioma (WHO grade II)	2	F	42	2	0	convexity		ben	N/A
MNG_241	Atypical meningioma (WHO grade II)	2	F	56	22	0	convexity		ben	N/A
MNG_242	Atypical meningioma (WHO grade II)	2	M	62	22	1	convexity		ben	N/A
MNG_243	Secretory meningioma (WHO grade I)	1	F	64	4	0	convexity		ben	N/A
MNG_244	Anaplastic meningioma (WHO grade III)	3	M	61	5	1	convexity		mal	N/A
MNG_245	Chordoid meningioma (WHO grade II)	2	F	49	29	0	N/A		ben	N/A
MNG_246	Chordoid meningioma (WHO grade II)	2	F	49	27	0	N/A		ben	N/A
MNG_247	Atypical meningioma (WHO grade II)	2	F	72	20	1	convexity		mal	N/A
MNG_248	Anaplastic meningioma (WHO grade III)	3	F	61	34	1	convexity		mal	N/A
MNG_249	Atypical meningioma (WHO grade II)	2	M	50	53	0	basal		int	N/A
MNG_250	Atypical meningioma (WHO grade II)	2	F	60	22	1	N/A		int	N/A
MNG_251	Secretory meningioma (WHO grade I)	1	F	78	27	0	basal		ben	N/A
MNG_252	Atypical meningioma (WHO grade II)	2	M	72	10	1	N/A		ben	N/A
MNG_253	Atypical meningioma (WHO grade II)	2	M	80	1	0	convexity		int	N/A
MNG_254	Atypical meningioma (WHO grade II)	2	F	60	86	0	N/A		ben	N/A
MNG_255	Anaplastic meningioma (WHO grade III)	3	F	53	13	1	basal		mal	N/A
MNG_256	Atypical meningioma (WHO grade II)	2	F	19	97	0	convexity		ben	N/A
MNG_257	Anaplastic meningioma (WHO grade III)	3	M	32	1	0	convexity		ben	N/A
MNG_258	Anaplastic meningioma (WHO grade III)	3	F	70	84	0	convexity		ben	N/A
MNG_259	Secretory meningioma (WHO grade I)	1	F	73	9	0	posterior fossa		ben	N/A
MNG_260	Psammomatous meningioma (WHO grade I)	1	F	67	6	0	spinal		int	N/A
MNG_261	Anaplastic meningioma (WHO grade III)	3	F	42	32	0	convexity		ben	N/A
MNG_262	Secretory meningioma (WHO grade I)	1	F	40	45	0	basal		ben	N/A
MNG_263	Atypical meningioma (WHO grade II)	2	F	45	59	0	convexity		ben	N/A

MNG_264	Secretory meningioma (WHO grade I)	1	F	51	18	0	basal		ben	N/A
MNG_265	Atypical meningioma (WHO grade II)	2	F	38	3	0	basal		ben	N/A
MNG_266	Secretory meningioma (WHO grade I)	1	F	65	84	1	basal		ben	N/A
MNG_267	Anaplastic meningioma (WHO grade III)	3	F	65	39	0	posterior fossa		int	N/A
MNG_268	Meningioma NOS	1	F	N/A	31	1	supratentorial		int	N/A
MNG_269	Chordoid meningioma (WHO grade II)	2	F	42	31	1	basal		ben	N/A
MNG_270	Meningioma NOS	1	F	58	26	0	convexity		ben	N/A
MNG_271	Transitional meningioma (WHO Grade I)	1	M	47	25	0	convexity		ben	N/A
MNG_272	Atypical meningioma (WHO grade II)	2	M	47	5	1	supratentorial		int	N/A
MNG_273	Meningioma NOS	1	F	54	29	0	convexity		ben	N/A
MNG_274	Transitional meningioma (WHO Grade I)	1	F	60	19	0	spinal		int	N/A
MNG_275	Meningioma NOS	1	F	61	5	0	convexity		int	N/A
MNG_276	Secretory meningioma (WHO grade I)	1	F	77	12	1	basal		ben	N/A
MNG_277	Atypical meningioma (WHO grade II)	2	F	65	3	0	basal		ben	N/A
MNG_278	Atypical meningioma (WHO grade II)	2	F	64	31	0	posterior fossa		ben	N/A
MNG_279	Meningioma NOS	1	M	76	21	0	convexity		ben	N/A
MNG_280	Transitional meningioma (WHO Grade I)	1	F	80	2	0	supratentorial		int	N/A
MNG_281	Transitional meningioma (WHO Grade I)	1	M	39	27	0	convexity		int	N/A
MNG_282	Atypical meningioma (WHO grade II)	2	M	39	27	0	convexity		int	N/A
MNG_283	Atypical meningioma (WHO grade II)	2	M	73	29	0	convexity		int	N/A
MNG_284	Meningioma NOS	1	M	57	18	1	posterior fossa		int	N/A
MNG_285	Atypical meningioma (WHO grade II)	2	F	61	24	1	convexity		int	N/A
MNG_286	Transitional meningioma (WHO Grade I)	1	F	67	30	0	convexity		int	N/A
MNG_287	Meningioma NOS	1	F	50	1	0	convexity		ben	N/A
MNG_288	Meningioma NOS	1	F	44	3	0	posterior fossa		ben	N/A
MNG_289	Transitional meningioma (WHO Grade I)	1	M	71	20	0	convexity		int	N/A
MNG_290	Atypical meningioma (WHO grade II)	2	M	34	12	0	N/A		ben	N/A
MNG_291	Atypical meningioma (WHO grade II)	2	F	36	6	0	basal		ben	N/A
MNG_292	Meningioma NOS	1	F	50	26	0	convexity		int	N/A
MNG_293	Meningioma NOS	1	M	60	4	0	convexity		int	N/A
MNG_294	Transitional meningioma (WHO Grade I)	1	F	54	15	0	supratentorial		ben	N/A
MNG_295	Meningioma NOS	1	M	69	24	0	convexity		int	N/A
MNG_296	Meningioma NOS	1	M	63	16	0	posterior fossa		ben	N/A
MNG_297	Atypical meningioma (WHO grade II)	2	M	65	7	1	convexity		int	N/A
MNG_298	Anaplastic meningioma (WHO grade III)	3	F	61	4	1	convexity		mal	N/A
MNG_299	Atypical meningioma (WHO grade II)	2	F	77	11	0	posterior fossa		ben	N/A
MNG_300	Meningioma NOS	1	M	57	10	0	basal		ben	N/A
MNG_301	Secretory meningioma (WHO grade I)	1	F	78	2	0	convexity		ben	N/A

MNG_302	Atypical meningioma (WHO grade II)	2	F	58	24	1	convexity		int	N/A
MNG_303	Transitional meningioma (WHO Grade I)	1	F	43	7	0	supratentorial		ben	N/A
MNG_304	Transitional meningioma (WHO Grade I)	1	M	36	20	0	supratentorial		ben	N/A
MNG_305	Atypical meningioma (WHO grade II)	2	F	40	13	0	convexity		ben	N/A
MNG_306	Meningioma NOS	1	M	64	11	0	basal		ben	N/A
MNG_307	Atypical meningioma (WHO grade II)	2	F	84	8	1	convexity		int	N/A
MNG_308	Atypical meningioma (WHO grade II)	2	F	68	26	0	convexity		ben	N/A
MNG_309	Atypical meningioma (WHO grade II)	2	F	41	24	0	convexity		int	N/A
MNG_310	Transitional meningioma (WHO Grade I)	1	F	55	10	0	convexity		ben	N/A
MNG_311	Atypical meningioma (WHO grade II)	2	F	56	18	0	convexity		int	N/A
MNG_312	Atypical meningioma (WHO grade II)	2	F	48	20	0	convexity		ben	N/A
MNG_313	Atypical meningioma (WHO grade II)	2	M	69	8	1	convexity		int	N/A
MNG_314	Atypical meningioma (WHO grade II)	2	M	82	1	0	posterior fossa		ben	N/A
MNG_315	Atypical meningioma (WHO grade II)	2	M	56	18	0	supratentorial		int	N/A
MNG_316	Atypical meningioma (WHO grade II)	2	F	70	17	0	convexity		int	N/A
MNG_317	Transitional meningioma (WHO Grade I)	1	M	72	1	0	convexity		int	N/A
MNG_318	Anaplastic meningioma (WHO grade III)	3	F	85	2	0	supratentorial		mal	N/A
MNG_319	Meningioma NOS	1	F	78	15	0	convexity		mal	N/A
MNG_320	Atypical meningioma (WHO grade II)	2	F	60	13	0	convexity		ben	N/A
MNG_321	Transitional meningioma (WHO Grade I)	1	M	44	20	1	convexity		int	N/A
MNG_322	Atypical meningioma (WHO grade II)	2	M	67	16	1	convexity		int	N/A
MNG_323	Atypical meningioma (WHO grade II)	2	M	78	26	0	convexity		ben	N/A
MNG_324	Atypical meningioma (WHO grade II)	2	M	21	22	0	convexity		mal	N/A
MNG_325	Atypical meningioma (WHO grade II)	2	F	68	7	0	convexity		int	N/A
MNG_326	Meningioma NOS	1	F	41	16	0	basal		ben	N/A
MNG_327	Meningioma NOS	1	M	68	15	0	convexity		ben	N/A
MNG_328	Meningioma NOS	1	F	47	15	0	basal		ben	N/A
MNG_329	Meningioma NOS	1	M	59	16	0	spinal		int	N/A
MNG_330	Meningioma NOS	1	F	45	14	0	spinal		int	N/A
MNG_331	Transitional meningioma (WHO Grade I)	1	F	56	20	0	spinal		ben	N/A
MNG_332	Transitional meningioma (WHO Grade I)	1	F	51	11	0	convexity		ben	N/A
MNG_333	Anaplastic meningioma (WHO grade III)	3	F	78	5	1	convexity		mal	N/A
MNG_334	Angiomatous meningioma (WHO Grade I)	1	F	67	6	0	convexity		ben	N/A
MNG_335	Atypical meningioma (WHO grade II)	2	M	76	11	0	basal		int	N/A
MNG_336	Atypical meningioma (WHO grade II)	2	F	48	15	0	basal		ben	N/A
MNG_337	Atypical meningioma (WHO grade II)	2	F	61	13	0	convexity		ben	N/A
MNG_338	Transitional meningioma (WHO Grade I)	1	F	53	15	0	supratentorial		ben	N/A
MNG_339	Atypical meningioma (WHO grade II)	2	F	33	19	0	convexity		int	N/A

MNG_340	Atypical meningioma (WHO grade II)	2	F	69	14	0	convexity		int	N/A
MNG_341	Atypical meningioma (WHO grade II)	2	F	67	12	0	convexity		ben	N/A
MNG_342	Anaplastic meningioma (WHO grade III)	3	M	80	1	1	supratentorial		int	N/A
MNG_343	Atypical meningioma (WHO grade II)	2	M	70	13	0	supratentorial		int	N/A
MNG_344	Atypical meningioma (WHO grade II)	2	F	54	15	0	convexity		int	N/A
MNG_345	Fibroblastic meningioma (WHO Grade I)	1	F	47	18	0	convexity		ben	N/A
MNG_346	Atypical meningioma (WHO grade II)	2	F	16	13	0	spinal		int	N/A
MNG_347	Atypical meningioma (WHO grade II)	2	F	33	20	0	posterior fossa		ben	N/A
MNG_348	Fibroblastic meningioma (WHO Grade I)	1	F	73	19	0	basal		ben	N/A
MNG_349	Meningioma NOS	1	M	49	11	0	convexity		int	N/A
MNG_350	Transitional meningioma (WHO Grade I)	1	M	74	36	1	convexity		int	N/A
MNG_351	Transitional meningioma (WHO Grade I)	1	F	40	22	1	basal		ben	N/A
MNG_352	Atypical meningioma (WHO grade II)	2	F	82	11	0	convexity		mal	N/A
MNG_353	Atypical meningioma (WHO grade II)	2	M	57	16	0	convexity		mal	N/A
MNG_354	Atypical meningioma (WHO grade II)	2	F	N/A	2	0	convexity		int	N/A
MNG_355	Atypical meningioma (WHO grade II)	2	F	28	12	0	supratentorial		int	N/A
MNG_356	Meningothelial meningioma (WHO Grade I)	1	F	35	11	0	basal		ben	N/A
MNG_357	Atypical meningioma (WHO grade II)	2	F	75	1	0	supratentorial		int	N/A
MNG_358	Atypical meningioma (WHO grade II)	2	F	74	4	0	convexity		int	N/A
MNG_359	Meningioma NOS	1	F	42	3	0	convexity		ben	N/A
MNG_360	Transitional meningioma (WHO Grade I)	1	F	66	7	0	convexity		int	N/A
MNG_361	Meningioma NOS	1	F	66	8	0	convexity		int	N/A
MNG_362	Meningioma NOS	1	F	59	9	0	supratentorial		int	N/A
MNG_363	Atypical meningioma (WHO grade II)	2	F	84	2	0	convexity		mal	N/A
MNG_364	Fibroblastic meningioma (WHO Grade I)	1	M	66	15	1	basal		int	N/A
MNG_365	Atypical meningioma (WHO grade II)	2	F	64	3	0	supratentorial		int	N/A
MNG_366	Meningioma NOS	1	F	78	1	0	convexity		ben	N/A
MNG_367	Meningioma NOS	1	M	16	9	0	convexity		int	N/A
MNG_368	Metaplastic meningioma (WHO Grade I)	1	F	65	11	0	convexity		ben	N/A
MNG_369	Atypical meningioma (WHO grade II)	2	F	56	7	0	convexity		mal	N/A
MNG_370	Atypical meningioma (WHO grade II)	2	M	85	9	0	basal		ben	N/A
MNG_371	Fibroblastic meningioma (WHO Grade I)	1	F	52	4	0	convexity		ben	N/A
MNG_372	Fibroblastic meningioma (WHO Grade I)	1	F	N/A	12	0	convexity		ben	N/A
MNG_373	Fibroblastic meningioma (WHO Grade I)	1	F	N/A	3	0	convexity		ben	N/A
MNG_374	Atypical meningioma (WHO grade II)	2	M	51	51	0	convexity		int	N/A
MNG_375	Transitional meningioma (WHO Grade I)	1	F	57	15	0	convexity		ben	N/A
MNG_376	Meningioma NOS	1	F	84	3	0	basal		ben	N/A
MNG_377	Atypical meningioma (WHO grade II)	2	F	58	11	0	convexity		int	N/A

MNG_378	Meningothelial meningioma (WHO Grade I)	1	M	56	45	1	convexity		int	N/A
MNG_379	Meningioma NOS	1	F	38	7	0	convexity		ben	N/A
MNG_380	Atypical meningioma (WHO grade II)	2	M	22	5	0	convexity		int	N/A
MNG_381	Meningioma NOS	1	M	52	2	0	convexity		ben	N/A
MNG_382	Meningioma NOS	1	M	71	1	0	supratentorial		ben	N/A
MNG_383	Meningioma NOS	1	M	61	3	0	convexity		int	N/A
MNG_384	Atypical meningioma (WHO grade II)	2	F	74	4	0	basal		int	N/A
MNG_385	Meningioma NOS	1	F	40	6	0	basal		ben	N/A
MNG_386	Meningioma NOS	1	M	53	7	0	posterior fossa		ben	N/A
MNG_387	Atypical meningioma (WHO grade II)	2	M	58	5	0	convexity		mal	N/A
MNG_388	Transitional meningioma (WHO Grade I)	1	M	82	2	0	convexity		ben	N/A
MNG_389	Meningioma NOS	1	M	75	3	0	spinal		ben	N/A
MNG_390	Atypical meningioma (WHO grade II)	2	M	35	5	0	convexity		int	N/A
MNG_391	Meningioma NOS	1	F	72	8	0	convexity		int	N/A
MNG_392	Atypical meningioma (WHO grade II)	2	M	70	3	0	basal		int	N/A
MNG_393	Atypical meningioma (WHO grade II)	2	F	49	11	0	convexity		int	N/A
MNG_394	Atypical meningioma (WHO grade II)	2	M	64	9	0	basal		int	N/A
MNG_395	Atypical meningioma (WHO grade II)	2	M	N/A	127	0	convexity		ben	wt
MNG_396	Atypical meningioma (WHO grade II)	2	F	N/A	122	0	convexity		ben	wt
MNG_397	Atypical meningioma (WHO grade II)	2	M	N/A	121	0	convexity		int	wt
MNG_398	Atypical meningioma (WHO grade II)	2	F	N/A	149	0	basal		mal	wt
MNG_399	Atypical meningioma (WHO grade II)	2	F	N/A	141	0	convexity		int	wt
MNG_400	Atypical meningioma (WHO grade II)	2	F	N/A	140	0	spinal		int	wt
MNG_401	Atypical meningioma (WHO grade II)	2	M	N/A	23	1	basal		int	wt
MNG_402	Atypical meningioma (WHO grade II)	2	F	N/A	140	0	basal		ben	wt
MNG_403	Atypical meningioma (WHO grade II)	2	M	N/A	27	1	convexity		int	wt
MNG_404	Atypical meningioma (WHO grade II)	2	M	N/A	128	0	convexity		int	wt
MNG_405	Atypical meningioma (WHO grade II)	2	M	N/A	16	0	convexity		int	wt
MNG_406	Atypical meningioma (WHO grade II)	2	M	N/A	21	1	convexity		int	wt
MNG_407	Atypical meningioma (WHO grade II)	2	F	N/A	15	1	basal		int	wt
MNG_408	Angiomatous meningioma (WHO Grade I)	1	M	61	39	0	convexity		ben	wt
MNG_409	Atypical meningioma (WHO grade II)	2	F	84	2	0	supratentorial		ben	wt
MNG_410	Atypical meningioma (WHO grade II)	2	F	69	45	0	N/A		int	wt
MNG_411	Transitional meningioma (WHO Grade I)	1	F	71	24	0	convexity		int	wt
MNG_412	Transitional meningioma (WHO Grade I)	1	F	58	33	0	N/A		int	wt
MNG_413	Atypical meningioma (WHO grade II)	2	F	60	51	0	N/A		ben	wt
MNG_414	Transitional meningioma (WHO Grade I)	1	F	64	51	0	convexity		ben	wt
MNG_415	Transitional meningioma (WHO Grade I)	1	F	62	54	0	N/A		int	wt

MNG_416	Atypical meningioma (WHO grade II)	2	F	65	39	0	basal		ben	wt
MNG_417	Chordoid meningioma (WHO grade II)	2	F	45	3	0	convexity		ben	wt
MNG_418	Transitional meningioma (WHO Grade I)	1	M	65	78	1	supratentorial		ben	N/A
MNG_419	Transitional meningioma (WHO Grade I)	1	M	63	76	1	basal		int	N/A
MNG_420	Angiomatous meningioma (WHO Grade I)	1	F	55	3	0	convexity		ben	wt
MNG_421	Meningothelial meningioma (WHO Grade I)	1	F	74	63	1	convexity		int	N/A
MNG_422	Atypical meningioma (WHO grade II)	2	F	31	39	1	supratentorial		int	wt
MNG_423	Transitional meningioma (WHO Grade I)	1	F	63	46	1	convexity		int	wt
MNG_424	Transitional meningioma (WHO Grade I)	1	F	50	2	1	supratentorial		int	N/A
MNG_425	Chordoid meningioma (WHO grade II)	2	F	44	31	0	convexity		ben	wt
MNG_426	Atypical meningioma (WHO grade II)	2	M	46	39	0	basal		int	wt
MNG_427	Atypical meningioma (WHO grade II)	2	F	47	3	0	convexity		ben	wt
MNG_428	Meningothelial meningioma (WHO Grade I)	1	F	51	291	0	supratentorial		ben	wt
MNG_429	Atypical meningioma (WHO grade II)	2	F	80	25	0	convexity		ben	wt
MNG_430	Meningothelial meningioma (WHO Grade I)	1	F	50	11	1	basal		ben	wt
MNG_431	Atypical meningioma (WHO grade II)	2	F	58	6	0	convexity		int	wt
MNG_432	Fibroblastic meningioma (WHO Grade I)	1	F	56	159	0	convexity		ben	wt
MNG_433	Microcystic meningioma (WHO grade I)	1	F	63	3	0	convexity		ben	wt
MNG_434	Transitional meningioma (WHO Grade I)	1	F	50	111	0	supratentorial		ben	wt
MNG_435	Chordoid meningioma (WHO grade II)	2	F	47	28	1	convexity		ben	wt
MNG_436	Fibroblastic meningioma (WHO Grade I)	1	F	51	49	0	convexity		ben	wt
MNG_437	Atypical meningioma (WHO grade II)	2	F	76	4	0	convexity		int	wt
MNG_438	Psammomatous meningioma (WHO grade I)	1	F	69	61	0	spinal		ben	wt
MNG_439	Psammomatous meningioma (WHO grade I)	1	F	73	46	0	spinal		int	wt
MNG_440	Psammomatous meningioma (WHO grade I)	1	F	47	59	0	convexity		ben	wt
MNG_441	Metaplastic meningioma (WHO Grade I)	1	M	66	13	0	convexity		ben	wt
MNG_442	Psammomatous meningioma (WHO grade I)	1	F	72	40	0	convexity		ben	wt
MNG_443	Metaplastic meningioma (WHO Grade I)	1	F	60	72	0	convexity		int	wt
MNG_444	Psammomatous meningioma (WHO grade I)	1	F	71	66	0	convexity		ben	wt
MNG_445	Psammomatous meningioma (WHO grade I)	1	M	29	67	0	spinal		int	wt
MNG_446	Meningothelial meningioma (WHO Grade I)	1	F	69	143	0	spinal		ben	wt
MNG_447	Transitional meningioma (WHO Grade I)	1	F	59	6	0	convexity		ben	wt
MNG_448	Meningothelial meningioma (WHO Grade I)	1	F	64	1	0	spinal		ben	wt
MNG_449	Meningothelial meningioma (WHO Grade I)	1	F	66	142	0	N/A		ben	wt
MNG_450	Angiomatous meningioma (WHO Grade I)	1	F	68	3	0	basal		ben	wt
MNG_451	Transitional meningioma (WHO Grade I)	1	F	54	56	0	convexity		ben	wt
MNG_452	Meningothelial meningioma (WHO Grade I)	1	F	59	2	0	spinal		ben	wt
MNG_453	Atypical meningioma (WHO grade II)	2	M	54	15	1	convexity		int	C228T

MNG_454	Transitional meningioma (WHO Grade I)	1	F	53	1	0	basal		ben	wt
MNG_455	Anaplastic meningioma (WHO grade III)	3	M	83	36	1	convexity		mal	wt
MNG_456	Microcystic meningioma (WHO grade I)	1	F	62	189	0	convexity		ben	wt
MNG_457	Microcystic meningioma (WHO grade I)	1	F	22	21	1	basal		ben	wt
MNG_458	Microcystic meningioma (WHO grade I)	1	F	56	150	0	convexity		int	wt
MNG_459	Transitional meningioma (WHO Grade I)	1	F	61	22	0	posterior fossa		ben	wt
MNG_460	Microcystic meningioma (WHO grade I)	1	M	50	186	0	convexity		int	wt
MNG_461	Transitional meningioma (WHO Grade I)	1	M	60	143	0	basal		ben	wt
MNG_462	Transitional meningioma (WHO Grade I)	1	M	71	70	0	basal		ben	wt
MNG_463	Metaplastic meningioma (WHO Grade I)	1	F	42	90	0	supratentorial		ben	wt
MNG_464	Metaplastic meningioma (WHO Grade I)	1	M	44	9	0	convexity		ben	wt
MNG_465	Transitional meningioma (WHO Grade I)	1	M	56	67	0	convexity		int	wt
MNG_466	Atypical meningioma (WHO grade II)	2	F	71	71	1	convexity		mal	N/A
MNG_467	Transitional meningioma (WHO Grade I)	1	F	55	28	0	posterior fossa		ben	wt
MNG_468	Transitional meningioma (WHO Grade I)	1	F	55	28	0	posterior fossa		ben	N/A
MNG_469	Rhabdoid meningioma (WHO grade III)	3	M	77	9	1	convexity		int	C250T
MNG_470	Atypical meningioma (WHO grade II)	2	M	75	28	0	convexity		mal	wt
MNG_471	Atypical meningioma (WHO grade II)	2	M	83	27	1	convexity		int	wt
MNG_472	Atypical meningioma (WHO grade II)	2	M	57	60	1	convexity		int	wt
MNG_473	Atypical meningioma (WHO grade II)	2	M	47	68	0	convexity		int	wt
MNG_474	Atypical meningioma (WHO grade II)	2	F	84	16	0	spinal		int	wt
MNG_475	Fibroblastic meningioma (WHO Grade I)	1	F	52	85	0	convexity		ben	wt
MNG_476	Atypical meningioma (WHO grade II)	2	F	56	2	0	convexity		ben	wt
MNG_477	Atypical meningioma (WHO grade II)	2	F	53	46	1	convexity		mal	wt
MNG_478	Fibroblastic meningioma (WHO Grade I)	1	F	52	49	0	convexity		int	wt
MNG_479	Atypical meningioma (WHO grade II)	2	F	64	48	1	convexity		mal	wt
MNG_480	Fibroblastic meningioma (WHO Grade I)	1	F	60	222	0	posterior fossa		ben	wt
MNG_481	Transitional meningioma (WHO Grade I)	1	M	59	42	0	convexity		ben	wt
MNG_482	Meningothelial meningioma (WHO Grade I)	1	M	66	84	0	convexity		ben	wt
MNG_483	Meningothelial meningioma (WHO Grade I)	1	F	57	60	0	basal		ben	wt
MNG_484	Meningothelial meningioma (WHO Grade I)	1	M	64	170	0	basal		ben	wt
MNG_485	Atypical meningioma (WHO grade II)	2	F	50	3	1	supratentorial		int	N/A
MNG_486	Chordoid meningioma (WHO grade II)	2	F	51	20	0	convexity		ben	wt
MNG_487	Atypical meningioma (WHO grade II)	2	F	79	23	1	convexity		int	wt
MNG_488	Atypical meningioma (WHO grade II)	2	F	38	65	0	posterior fossa		ben	wt
MNG_489	Atypical meningioma (WHO grade II)	2	M	66	77	1	convexity		int	wt
MNG_490	Transitional meningioma (WHO Grade I)	1	F	62	12	0	N/A		ben	wt
MNG_491	Metaplastic meningioma (WHO Grade I)	1	F	46	4	0	convexity		ben	wt

MNG_492	Meningothelial meningioma (WHO Grade I)	1	F	67	55	1	basal		ben	wt
MNG_493	Fibroblastic meningioma (WHO Grade I)	1	F	64	39	0	posterior fossa		ben	wt
MNG_494	Fibroblastic meningioma (WHO Grade I)	1	F	55	4	0	convexity		ben	wt
MNG_495	Fibroblastic meningioma (WHO Grade I)	1	F	45	15	1	basal		int	wt
MNG_496	Angiomatous meningioma (WHO Grade I)	1	F	56	18	0	posterior fossa		ben	wt
MNG_497	Fibroblastic meningioma (WHO Grade I)	1	F	76	11	0	N/A		ben	wt
MNG_498	Angiomatous meningioma (WHO Grade I)	1	F	46	7	0	spinal		int	wt
MNG_499	Fibroblastic meningioma (WHO Grade I)	1	F	78	65	0	convexity		ben	wt
MNG_500	Angiomatous meningioma (WHO Grade I)	1	F	61	15	0	basal		int	wt
MNG_501	Transitional meningioma (WHO Grade I)	1	M	53	35	0	convexity		int	wt
MNG_502	Transitional meningioma (WHO Grade I)	1	M	40	35	0	convexity		int	wt
MNG_503	Meningothelial meningioma (WHO Grade I)	1	F	64	7	0	supratentorial		ben	wt
MNG_504	Angiomatous meningioma (WHO Grade I)	1	F	73	39	0	convexity		ben	wt
MNG_505	Anaplastic meningioma (WHO grade III)	3	M	45	4	1	convexity		mal	wt
MNG_506	Anaplastic meningioma (WHO grade III)	3	F	69	39	0	convexity		int	wt
MNG_507	Anaplastic meningioma (WHO grade III)	3	M	63	1	1	convexity		mal	wt
MNG_508	Anaplastic meningioma (WHO grade III)	3	M	71	28	1	posterior fossa		mal	wt
MNG_509	Anaplastic meningioma (WHO grade III)	3	F	74	16	1	convexity		mal	wt
MNG_510	Anaplastic meningioma (WHO grade III)	3	F	66	12	1	convexity		mal	wt
MNG_511	Anaplastic meningioma (WHO grade III)	3	F	60	78	1	convexity		mal	wt
MNG_512	Anaplastic meningioma (WHO grade III)	3	M	69	108	0	convexity		int	wt
MNG_513	Anaplastic meningioma (WHO grade III)	3	M	72	3	0	convexity		mal	wt
MNG_514	Anaplastic meningioma (WHO grade III)	3	F	56	32	1	convexity		mal	wt
MNG_515	Anaplastic meningioma (WHO grade III)	3	F	65	13	1	convexity		mal	C250T
MNG_516	Anaplastic meningioma (WHO grade III)	3	M	74	1	1	supratentorial		int	wt
MNG_517	Anaplastic meningioma (WHO grade III)	3	M	67	10	1	convexity		mal	wt
MNG_518	Anaplastic meningioma (WHO grade III)	3	M	60	12	0	convexity		mal	wt
MNG_519	Anaplastic meningioma (WHO grade III)	3	M	59	26	1	convexity		mal	wt
MNG_520	Meningioma NOS	1	F	81	39	0	convexity		int	wt
MNG_521	Meningioma NOS	1	M	47	11	1	convexity		ben	wt
MNG_522	Transitional meningioma (WHO Grade I)	1	F	22	53	0	N/A		int	wt
MNG_523	Secretory meningioma (WHO grade I)	1	M	65	13	0	convexity		ben	wt
MNG_524	Atypical meningioma (WHO grade II)	2	M	59	7	1	convexity		int	wt
MNG_525	Clear cell meningioma (WHO grade II)	2	F	16	51	0	posterior fossa		int	wt
MNG_526	Clear cell meningioma (WHO grade II)	2	F	16	23	1	posterior fossa		int	N/A
MNG_527	Clear cell meningioma (WHO grade II)	2	F	27	78	1	posterior fossa		int	wt
MNG_528	Clear cell meningioma (WHO grade II)	2	M	34	8	0	spinal		int	wt

Suppl. Table 3 – Prognostic associations (multivariable analysis)

		Hazard Ratio	95% CI	p-value
**Age**	per 10 years increase	0.93	[0.82, 1.05]	0.25
**Sex**	male vs female	1.12	[0.81, 1.53]	0.49
**Location**	convexity vs basal	0.76	[0.50, 1.16]	0.20
	posterior fossa vs basal	0.54	[0.26, 1.12]	0.10
	spinal vs basal	0.54	[0.16, 1.80]	0.32
	supratentorial vs basal	1.35	[0.64, 2.83]	0.43
**WHO grade**	II vs I	1.54	[1.02, 2.33]	0.04
	III vs I	3.09	[1.82, 5.22]	< 0.0001
**MC**	int vs ben	4.31	[2.69, 6.91]	< 0.0001
	mal vs ben	9.04	[4.93, 16.57]	< 0.0001
**CDKN2A/B homozy del**	yes vs no	2.06	[1.20, 3.53]	0.0089

Cox regression model, n = 528, number of events = 175. including WHO grade and methylation subtype.

Suppl. Fig. 1 – Time to progression or recurrence (TTP) of the 528 patients in the cohort stratified for *CDKN2A/B* homozygous deletion.

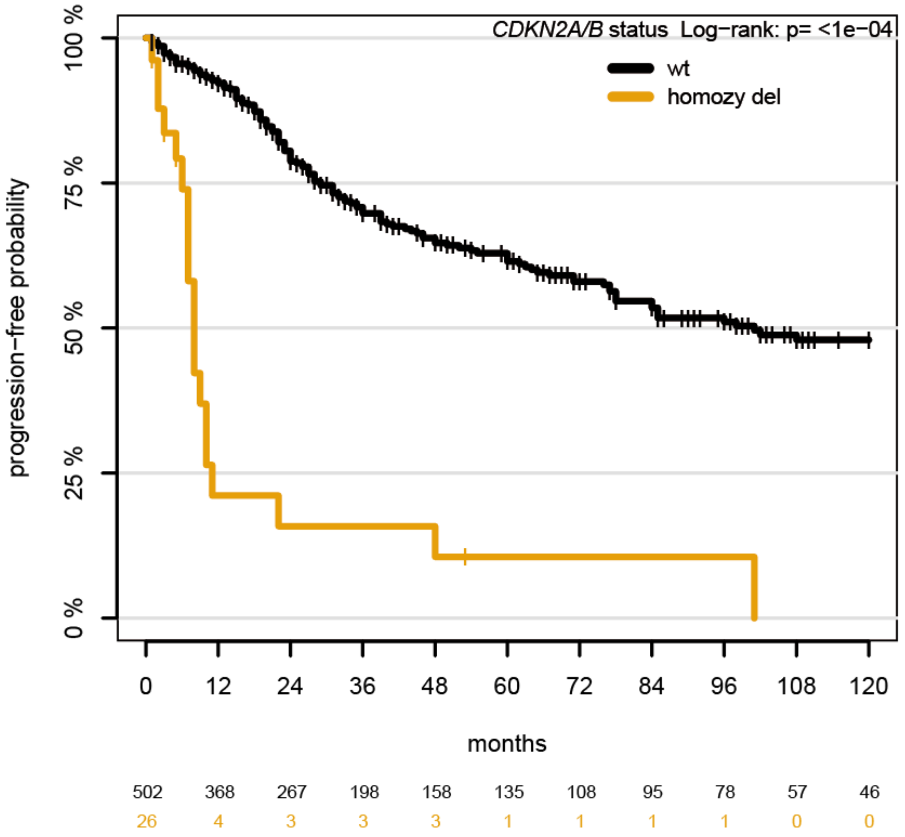

Suppl. Fig. 2 – Time to progression or recurrence (TTP) of the 293 patients in the cohort stratified for *TERT* promoter mutation status.

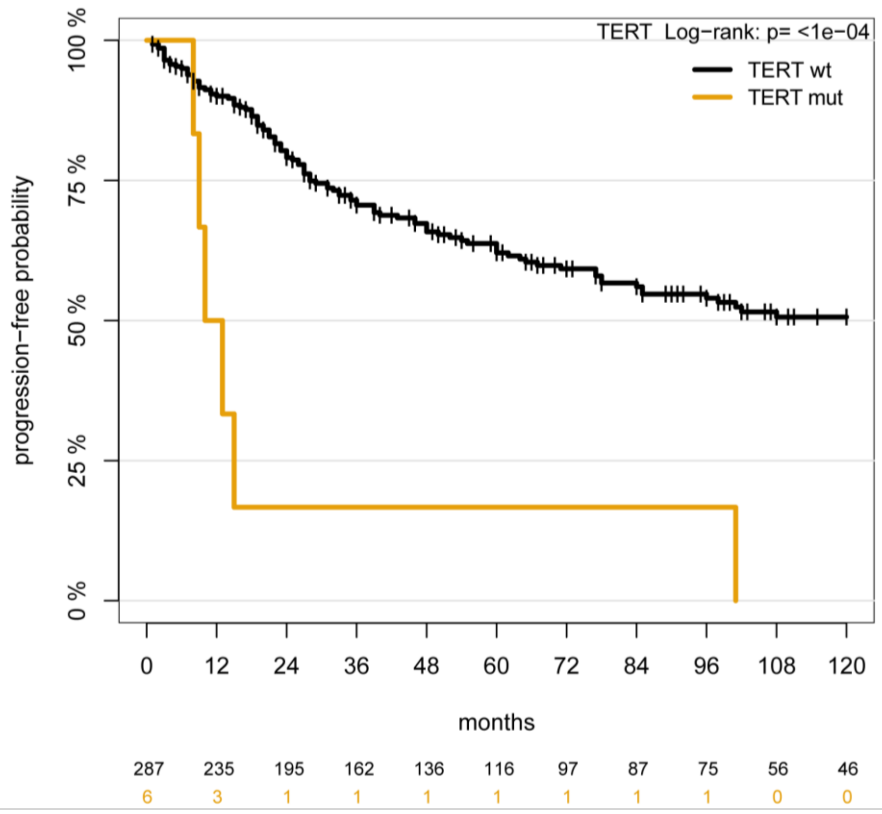

Suppl. Fig. 3 – Time to progression or recurrence (TTP) of the 528 patients in the cohort stratified for *CDKN2A/B* homozygous deletion vs methylation class (a, b).

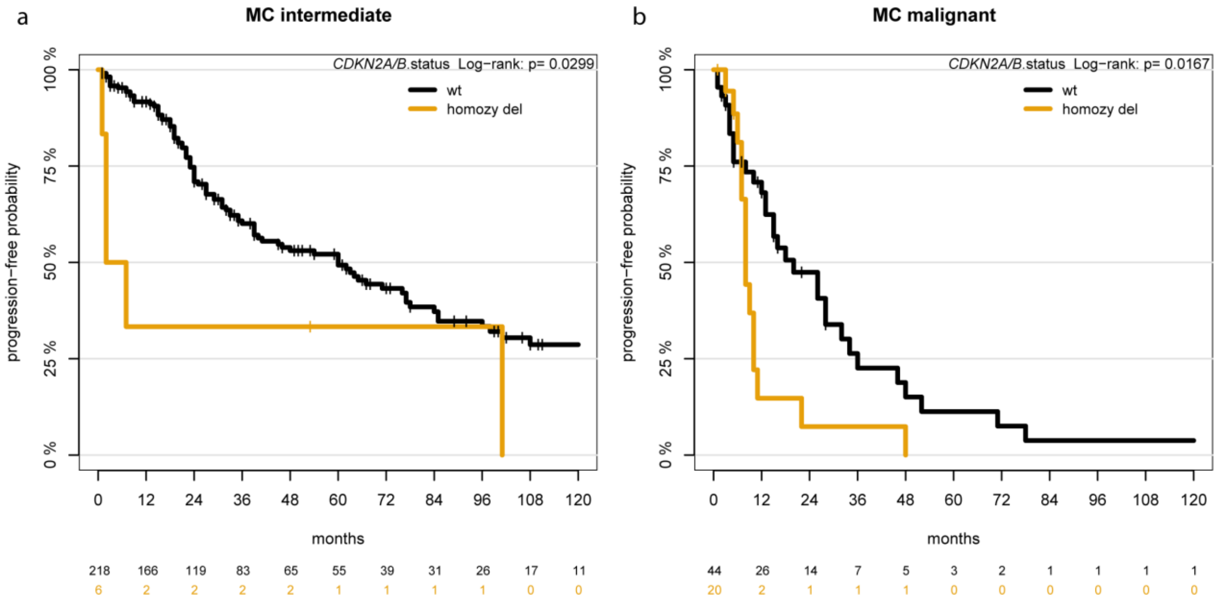

Supplement: Supplementary file 1 — Supplementary material 1 (PDF 896 kb) [file 401_2020_2188_MOESM1_ESM.pdf]
